# Dietary Magnesium Intake Affects the Association Between Serum Vitamin D and Type 2 Diabetes: A Cross-Sectional Study

**DOI:** 10.3389/fnut.2021.763076

**Published:** 2021-11-25

**Authors:** Weichao Huang, Xiaoman Ma, Hualiang Liang, Haojia Li, Jiayu Chen, Liujia Fang, Qilin Yang, Zhenhui Zhang

**Affiliations:** ^1^The Second Clinical College, Guangzhou Medical University, Guangzhou, China; ^2^The Third Clinical College, Guangzhou Medical University, Guangzhou, China; ^3^Department of Critical Care, The Second Affiliated Hospital of Guangzhou Medical University, Guangzhou, China

**Keywords:** magnesium, vitamin D, 25(OH)D, vitamin D (PubChem CID: 5280793) 2, vitamin D_3_, type 2 diabetes (T2DM) risk, dietary intake

## Abstract

**Introduction:** Circulating vitamin D concentrations have been associated with the risk of type 2 diabetes (T2D). Magnesium has also been reported to be associated with lower T2D risk. Besides, magnesium is an essential cofactor for vitamin D activation. However, the effect of dietary magnesium intake on the association between vitamin D and the risk of T2D has not been studied comprehensively. Therefore, we designed this cross-sectional study to assess the effect modification of magnesium intake on the association between vitamin D and risk of T2D.

**Research Design and Methods:** The present study analyzed data from the National Health and Nutrition Examination Survey (NHANES) continuously from 2007 to 2014, involving 10,249 participants. By having stratified participants based on magnesium intake category (low magnesium intake <267 mg/day; high magnesium intake: ≥267 mg/day), we further evaluated the difference (interaction test) between the relationship of vitamin D with the risk of T2D among low magnesium intake participants and high magnesium intake participants using weighted multivariable logistic regression.

**Results:** In this cross-sectional study, the association of serum vitamin D with the incidence of T2D appeared to differ between the low magnesium intake group and the high magnesium intake group (OR: 0.968, 95%Cl: 0.919–1.02 vs. OR: 0.925, 95%Cl: 0.883–0.97). Furthermore, there was evidence of interaction between vitamin D levels and magnesium intake on decreasing the incidence of T2D (*p*-value for interaction = 0.001).

**Conclusions:** The results of our study indicated that magnesium intake might affect the association of serum vitamin D with the risk of T2D. Such a finding requires further randomized controlled trials to provide more evidence.

## Introduction

The increasing prevalence of type 2 diabetes (T2D) has become a severe public health problem, leading to growth in related morbidity and mortality, apart from imposing a substantial economic burden (1). Therefore, it is essential to identify the nutritional components associated with diabetes to prevent T2D. Vitamin D deficiency is becoming a common health issue (2). It is universally acknowledged that vitamin D is primarily associated with calcium homeostasis (3). Furthermore, vitamin D deficiency can lead to systemic disorder of calcium and phosphorus metabolism, which contributes to bone-deforming rickets in children and osteoporosis in adults (3).

However, the relationship between vitamin D and T2D remains controversial. Several recent studies (4–7) have pointed to a possible association between vitamin D and the risk of T2D. A cohort study conducted by Pittas indicated that vitamin D intake played a potentially beneficial role in reducing the risk of T2D (8). Nevertheless, the study of Zhang showed that increasing 25(OH)D concentrations may not reduce the risk of T2D (9). Furthermore, the study conducted by Nina O. Nielsen did not find a significant association between low vitamin D levels and increased risk of T2D (10). The differences in the results of these studies may be attributed to potential confounding factors which have not been fully considered, such as dietary magnesium intake.

Magnesium is an essential cofactor for enzymes involved in glucose metabolism (11). One previous study has shown a high intake of magnesium being associated with reduced diabetes risk (12). A review demonstrated that magnesium interacts with vitamin D by activating the synthesis of 25(OH)D and 1,25(OH)2D and increasing the transfer to target tissues by elevating the vitamin D binding protein (VDBP) (13). However, limited clinical research has been performed investigating the effect of magnesium intake on the association between vitamin D and the risk of T2D. Therefore, in this cross-sectional study, we hypothesized that magnesium and vitamin D have an interactive effect on the risk of T2D. We aimed to explore the effect of magnesium intake on the association between vitamin D and the risk of T2D.

## Methods

### Data Sources and Study Population

This was a cross-sectional study. We used data from the National Health and Nutrition Examination Survey (NHANES) continuously from 2007 to 2014. Participants who were above 20 years of age and completed the interview and examination in the mobile examination center (MEC) were enrolled in our study. Participants with missing data on serum vitamin D concentration, covariates, and T2D status were excluded. NHANES is a health-related program of studies designed to assess the health and nutritional status of non-institutionalized US citizens. Survey participants were selected using a multistage, stratified probability design as a representative sample (14). An extensive household interview was performed to collect demographic and health history information. Physical examinations were conducted, and blood samples were collected in a MEC. The serum specimens were analyzed at the Division of Laboratory Sciences at the National Center for Environmental Health, Centers for Disease Control and Prevention. The samples were analyzed in America.

The study was approved by the National Center for Health Statistics Research Ethics Review Board. The original study protocol is accessible on the website of the Ethics Review Board of the National Center for Health Statistics Research (https://www.cdc.gov/nchs/nhanes/irba98.htm), duly approved by the ethical review committee (protocol #2005-06; #2011-17). Our study was based on the public data from NHANES and all details are from the official website (https://www.cdc.gov/nchs/nhanes/about_nhanes.htm).

### Measurement and Classification of 25(OH)D Concentrations

Blood specimens for measurement of 25(OH)D status collected during the MEC examination were processed and stored at −30°C until shipping to the CDC Environment Health Laboratory in Atlanta, Georgia. Serum samples were analyzed for 25-hydroxyvitamin D_3_[25(OH)D_3_], 25-hydroxyvitamin D_2_[25(OH)D_2_], and 3-epi-25-hydroxyvitamin D_3_[3-epi-25(OH)D_3_] concentrations using ultra-high-performance liquid chromatography-tandem mass spectrometry method (UHPLC-MS/MS). Total vitamin D status is defined as the sum of 25(OH)D_3_ and 25(OH)D_2_. Total vitamin D concentration was classified as deficient group (<50 nmol/L), suboptimal group (50–75 nmol/L), and sufficient group (>75 nmol/L), as suggested by the U.S. Endocrine Society (15).

### Magnesium Intake

Data regarding magnesium dietary intake for the previous 24-h period was collected through a dietary recall interview at the MEC. The daily magnesium intake was categorized as high or low intake based on the median value (267 mg/d). The 24-h recall method is most often used for determining dietary intake in large-scale surveys. The decision to continue with this method over the years in NHANES has been based on the consensus of expert groups during workshops held periodically to evaluate data collection methods in NHANES (16).

### Identification of T2D

The T2D case definition was based on the American Diabetes Association criteria (17) and a self-report questionnaire. Participants who fulfilled the following criteria were identified as T2D cases: (1) FPG ≥7 mmol/L, (2) HbA1c ≥6.5%, (3) 2-h plasma glucose ≥11.1 mmol/L during an oral glucose tolerance test, (4) self-report questionnaire data indicating physician diagnosis of diabetes, and current use of insulin or diabetes pill to lower blood glucose.

### Covariates

The present study considered the age, gender, race/ethnicity, the season of examination, physical activity, educational level, smoking status, body mass index (BMI), family income, and dietary intake including energy, protein, fiber, vitamin D, calcium, and magnesium of the participants as potential confounders. Race and ethnicity were categorized as Mexican American, other Hispanic, non-Hispanic white, non-Hispanic black, and other races. Educational level was categorized as did not graduate from high school, high school graduation, and college education or above. Smoking status was categorized as current smokers, former smokers, and never smokers. Participants who had smoked more than 100 cigarettes in the past and reported smoking either some days or every day at the time of the interview were considered to be current smokers. Participants who had smoked more than 100 cigarettes during their lifetime but did not smoke currently were considered former smokers. Participants who reported not having smoked even 100 cigarettes during their lifetime were considered never smokers. According to a standardized protocol, BMI was calculated from weight and height. The season was classified as winter or summer, depending upon whether the period of examination was between November to April or May to October, respectively. Physical activity was categorized based on three-level activity intensity, namely walking and moderate and vigorous activities. BMI was calculated from the measured height and body mass. Body mass was measured on a digital scale (Toledo Scale; Mettler-Toledo, LLC, Columbus, OH, USA) in pounds and converted to kilograms. Height was measured using an electronic stadiometer (Seca Ltd., Medical Scales and Measurement Systems, Birmingham, UK) to the nearest millimeter.

The dietary recall interview was performed before the interview at MEC to collect the previous 24-h dietary information, including total dietary food energy, vitamin D, calcium, magnesium, protein, and fiber.

We used the poverty income ratio (PIR), calculated by the family size-specific threshold, to define family income. Respondents who answered yes to the following question were classified as being at risk of diabetes: “Have you ever been told by a doctor or other health professional that you have health conditions or a medical or family history that increases your risk of diabetes?”

### Statistical Analysis

All the analyses were performed with the statistical software packages R (http://www.R-project.org, The R Foundation), Free Statistics software version 1.3 (18), and EmpowerStats (http://www.empowerstats.com). Comparisons of serum vitamin D levels in participants with diabetes and participants without diabetes were performed between low magnesium intake and high magnesium intake. We used the sample weight provided by NHANES. The subgroup analyses by magnesium intake were performed using the stratified weighted multivariate logistic regression model. The sensitivity analysis was performed by excluding the outliers, whose serum vitamin D value was out of the range of mean ± 2SD (0–143.2 nmol/L) or mean ± 3SD (9.2–118.7 nmol/L). Odds ratios (ORs) and 95% CIs were calculated. Interaction among subgroups was inspected by the likelihood ratio test. Tests for linear trend by entering the median value of each category of serum 25(OH)D as a continuous variable in the models were performed. *P*-value was considered statistically significant at <0.05.

The weighted means and SE for continuous variables, as well as the percentages (%) and SE for categorical variables, were present in the descriptive analysis. Categorical variables were analyzed by fitting a logistic regression model and computing a Wald-type interval on the log-odds scale (categorical variables). Continuous variables were analyzed by using the *t*-test (normal distribution), Kruskal–Wallis (skewed distribution) test, respectively.

## Results

### Baseline Characteristics of the Study Population

**Figure 1:**
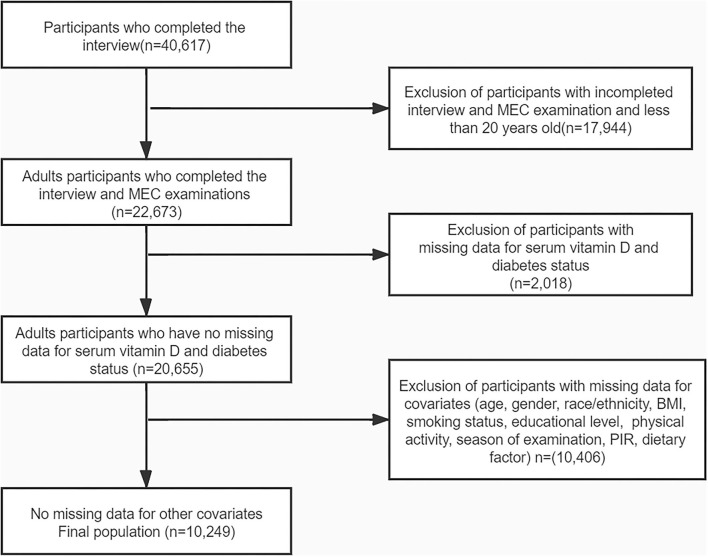
The flow chart of the study.

Four cycles of NHANES, 2007–2008, 2009–2010, 2011–2012, and 2013–2014, were used in this study. We identified 40,617 potential participants; 22,673 adults (≥20 years old) who completed the interview; and the MEC examination were enrolled in our study. Participants with missing data on serum 25-hydroxyvitamin D concentration (*n* = 2,018) were excluded. After excluding participants with missing data for covariates, the remaining 10,249 participants were included in our analysis. The flowchart of the exclusion criteria is depicted in Figure 1. As illustrated in the flow chart (Figure 1), a total of 10,249 subjects were included in the final analyses. Table 1 displays the descriptive characteristics of this study population according to dietary magnesium intake. Compared with the low magnesium intake individuals (<267 mg/day), those with a high intake of magnesium (≥267 mg/day) were more likely to be men, younger, well-educated, and mostly had a higher income. Individuals with high magnesium intake had lower values of 2-h glucose (2hOGTT) and a lower BMI. For dietary factors, intake of energy, protein, fiber, calcium, magnesium, and vitamin D was lower in participants whose intake of magnesium was low. Season of examination, fasting glucose, smoking habits, physical activities, and risk for diabetes did not differ by magnesium intake.

**Table 1:** Baseline characteristics of participants.

**Covariates**	**Dietary magnesium intake (mg/d)**			
	**Total** **(*n* = 10,249)**	** <267mg/d** **(*n* = 5,094)**	**≥267mg/d** **(*n* = 5,155)**	***p*-value**
Age (years)	47.1 ± 0.3	47.6 ± 0.4	46.7 ± 0.4	0.0458
**Gender**, ***n*** **(%)**				
Male	4,968 (47.7± 0.8)	2,366 (36 ± 1)	2,602 (57.7 ± 1)	<0.0001
Female	5,281 (52.3 ± 0.8)	3,595 (64 ±1)	1,686 (42.3 ± 1)	<0.0001
**Race/Ethnicity**, ***n*** **(%)**				
Mexican American	1,470 (8.1 ± 0.9)	757 (7.2 ± 1)	713 (8.9 ± 1)	0.0212
Other Hispanic	1,005 (5.7 ± 0.7)	593 (5.9 ± 0.8)	412 (5.4 ± 0.7)	0.3251
Non-Hispanic white	4,738 (69 ± 1.8)	2,688 (66.0 ± 2.1)	2,050 (71.6 ± 1.8)	0.0001
Non-Hispanic black	2,065 (10.5 ± 0.9)	1,399 (13.5 ± 1.2)	666 (7.8 ± 0.7)	<0.001
Other races	971 (6.8 ± 0.5)	524 (7.3± 0.6)	447 (6.3 ± 0.6)	0.0398
BMI (kg/m^2^), Mean ± SD	28. ± 0.1	29.3 ± 0.1	28.6 ± 0.2	0.0005
**Obesity**, ***n*** **(%)**				
No	6,449 (64.1 ± 0.7)	3,610 (61 ± 0.9)	2,839 (66.8 ± 1.1)	0.0002
Yes	3,800 (35.9 ± 0.7)	2,351 (39 ± 0.9)	1,449 (33.2 ± 1.1)	0.0002
**Season of examination**, ***n*** **(%)**				
Winter	4,812 (42.1 ± 3.2)	2,799 (43.3 ± 3.3)	2,013 (41.1 ± 3.4)	0.2899
Summer	5,437 (57.9 ± 3.2)	3,162 (56.7 ± 3.3)	2,275 (58.9 ± 3.4)	0.2899
Two Hour Glucose (OGTT) (mmol/L)	6.5 ± 0	6.6 ± 0.1	6.3 ± 0.1	0.0006
Fasting Glucose (mmol/L)	5.8 ± 0	5.8 ± 0	5.8 ± 0.1	0.9606
Glycohemoglobin (%)	5.6 ± 0	5.6 ± 0	5.6 ± 0	0.0421
**Smoking status**, ***n*** **(%)**				
Current smoker	2,202 (21.4 ± 0.6)	1,292 (21.9± 0.8)	910 (21 ± 0.8)	0.4879
Former smoker	2,483 (24.5 ± 0.7)	1,441 (24 ± 0.9)	1,042 (25 ± 0.8)	0.409
Never smoker	5,564 (54.1 ± 0.6)	3,228 (54.1 ± 0.9)	2,336 (54 ± 0.8)	0.9149
**Physical activity**, ***n*** **(%)**				
Vigorous work activity	1,836 (18 ± 0.5)	1,051 (17.5 ± 0.7)	785 (18.4 ± 0.7)	0.3272
Moderate work activity	2,157 (21.2 ± 0.6)	1,247 (21.5 ± 0.9)	910 (20.9 ± 0.8)	0.6154
Walk or bicycle	1,441 (13.8 ± 0.5)	838 (13.5 ± 0.7)	603 (13.9 ± 0.7)	0.6871
Vigorous recreational activities	682 (6.2 ± 0.3)	385 (5.7 ± 0.5)	297 (6.7 ± 0.4)	0.1473
Moderate recreational activities	4,133 (40.9 ± 0.7)	2,440 (41.8 ± 1.1)	1,693 (40.1 ± 1.1)	0.3049
**Education level**, ***n*** **(%)**				
Did not graduate from high school	2,563 (17.0 ± 0.9)	1,655 (19.8 ± 1.0)	908 (14.5 ± 0.9)	<0.0001
Graduated from high school	2,284 (22.0 ± 0.8)	1,447 (25.3 ± 1.0)	837 (19.1 ± 1.0)	<0.0001
College education or above	5,393 (61.0 ± 1.3)	2,852 (54.8 ± 1.3)	2,541 (66.4 ± 1.6)	<0.0001
PIR, *n* (%)	2.9 ± 0.1	2.7 ± 0.1	3.2 ± 0.1	<0.0001
**Dietary factors**				
Energy (kcal)	2,176.8 ± 14.5	1,627.5 ± 15.3	2,650.9 ± 20.7	<0.0001
Protein (gm)	84 ± 0.7	59.8± 0.6	104.9 ± 1	<0.0001
Fiber (gm)	17.3 ± 0.2	10.8 ± 0.1	22.9 ± 0.3	<0.0001
Magnesium (mg)	309.6 ± 3.1	191.7 ± 12	411.5 ± 2.5	<0.0001
Calcium (mg)	985.9 ± 10.9	668 ± 7.9	1,260.4.2 ± 12.8	<0.0001
Vitamin D (mg)	4.8 ± 0.1	2.9± 0.1	6.4 ± 0.2	<0.0001
**Risk for diabetes**				
Yes	1,171 (12.9 ± 0.5)	697 (12.4 ± 0.7)	474 (11.3 ± 0.7)	0.3016
No	7,839 (86.9 ± 0.5)	4,478 (78 ± 0.8)	33,661 (80.5 ± 0.9)	0.0659
Unknown	28 (0.2 ± 0.1)	16 (0.2 ± 0.1)	12 (0.2 ± 0.1)	0.8204
**T2D**, ***n*** **(%)**				
No	8,447 (86.8 ± 0.5)	4,810 (85.5 ± 0.6)	3,637 (87.9 ± 0.8)	0.0096
Yes	1,802 (13.2 ± 0.5)	1,151 (14.5 ± 0.6)	651 (12.1 ± 0.8)	0.0096

*PIR, ratio of family income to poverty; BMI, body mass index; T2D, type 2 diabetes*.

### Distribution of Serum Vitamin D in the T2D Group by Magnesium Intake

**Figure 2:**
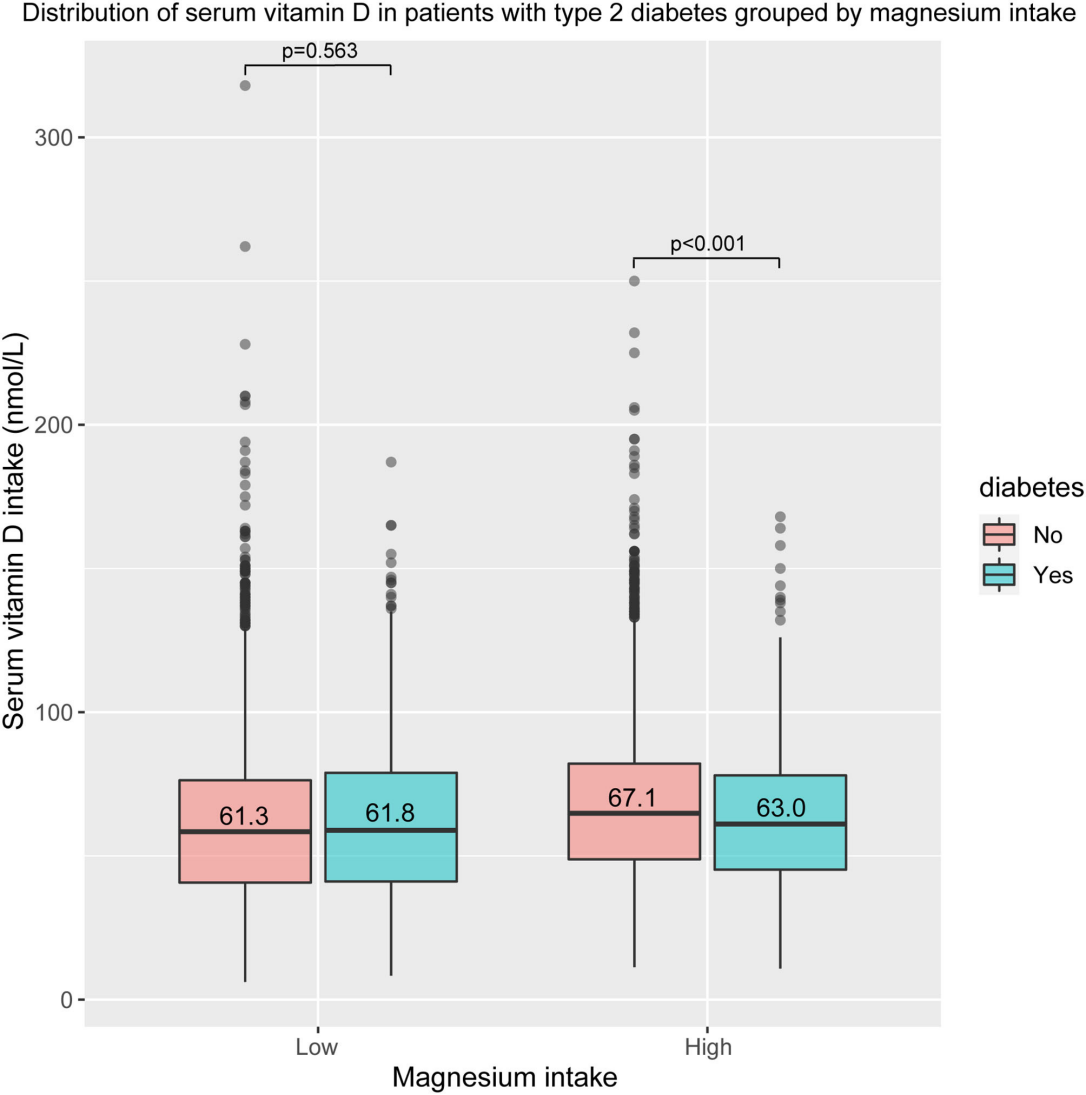
Distribution of serum vitamin D in patients with type 2 diabetes (T2D) group by magnesium intake.

Figure 2 shows the difference in serum vitamin D levels between T2D participants and non-T2D participants. It is not significant in the low magnesium intake group (61.3 vs. 61.8 nmol/L, *p* = 0.563). However, in the high magnesium intake group, serum vitamin D level in participants with T2D was significantly decreased, compared with non-T2D participants (67.1 vs. 63 nmol/L, *p* < 0.001). After excluding participants whose serum vitamin D value was >143.2 nmol/L, the finding remains stable. As shown in Supplementary Figure S1, there is still a significant difference in the high magnesium intake group (66 vs. 62.4, *p* < 0.001), but not in the low magnesium intake group (60.2 vs. 61.7, *p* = 0.084). After excluding participants whose vitamin D value was <9.2 nmol/L or >118.7 nmol/L, the finding is still significant (Supplementary Figure S2). The difference in the high magnesium intake group was significant (64 vs. 61.3, *p* = 0.002), while it is still non-significant in the low magnesium intake group (58.3 vs. 59.3, *p* = 0.212).

### Magnesium Intake Affects the Association Between Vitamin D and Risk of T2D

The univariate analysis indicated that age, gender, physical activity, PIR, obesity, education level, and dietary calcium intake were associated with T2D (Table 2).

**Table 2:** Association of covariates and T2D risk.

**Covariate**	**OR (95% Cl)**	***P*-value**
Age (years)	0.010 (0.008–0.012)	<0.001
**Gender**, ***n*** **(%)**		
Male	1 (reference)	
Female	0.798 (0.704–0.904)	<0.001
**Race/ethnicity**, ***n*** **(%)**		
Mexican American	1 (reference)	
Other Hispanic	0.096 (0.657–1.253)	0.555
Non-Hispanic white	0.766 (0.602–0.974)	0.034
Non-Hispanic black	1.170 (0.908–1.508)	0.229
Other races	0.973 (0.707–1.339)	0.866
**Obesity**, ***n*** **(%)**		
No	1 (reference)	
Yes	3.403 (2.870–4.035)	<0.001
**Education level**, ***n*** **(%)**		
Did not graduate from high school	1 (reference)	
Graduated from high school	0.671 (0.534–0.842)	0.001
College education or above	0.486 (0.402–0.589)	<0.001
**Smoking status**, ***n*** **(%)**		
Current smoker	1 (reference)	
Former smoker	1.097 (0.859–1.402)	0.462
Never smoker	1.118 (0.928–1.347)	0.244
**Physical activity**, ***n*** **(%)**		
Vigorous work activity	1 (reference)	
Moderate work activity	1.284 (0.989–1.667)	0.066
Walk or bicycle	1.846 (1.444–2.360)	<0.001
Vigorous recreational activities	1.675 (1.102–2.545)	0.019
Moderate recreational activities	1.166 (0.919–1.479)	0.211
PIR, n (%)	0.907 (0.869–0.947)	<0.001
**Season of examination**, ***n*** **(%)**		
Winter	1 (reference)	
Summer	0.931 (0.784–1.105)	0.417
Calcium intake (mg)	0.9997 (0.9996–0.9999)	0.002

*Data presented are ORs and 95% Cls. PIR, the ratio of family income to poverty*.

After adjusting for age, gender, race/ethnicity, obesity, education level, physical activity, smoking status, PIR, the season of examination, dietary calcium, magnesium, magnesium has a significant interaction effect on the association between vitamin D and risk of T2D (Table 3). The interaction also exists in the other three different adjustment models (Supplementary Tables S1–S3).

**Table 3:** Interactive effect of vitamin D and dietary magnesium intake on T2D (All participants).

**Variable**	**Low-magnesium intake**		**High-magnesium intake**		***P* for interaction**
	**(*****n*** **= 5,094)**		**(*****n*** **= 5,155)**		
	**OR (95% CI)**	***P*-value**	**OR (95% CI)**	***P-*value**	
Vitamin D (nmol/L)	0.968 (0.919–1.020)	0.225	0.925 (0.883–0.970)	0.002	0.001
Subgroups				<0.001	
Deficient (<50 nmol/L)	1 (reference)		1 (reference)		
Suboptimal (50–75 nmol/L)	0.843 (0.590–1.205)	0.354	0.683 (0.519–0.899)	0.009	
Sufficient (>75 nmol/L)	0.877 (0.596–1.289)	0.507	0.647 (0.462–0.907)	0.015	
Trend test		0.519		0.028	

*Adjusted for age, gender, race/ethnicity, obesity, education level, physical activity, smoking status, PIR, the season of examination, and dietary calcium intake*.

As total vitamin D increased, the incidence of T2D in the high magnesium intake group was significantly reduced (*p* = 0.002); that is, the interaction of magnesium intake on the association of vitamin D with T2D incidence was significant (the *p*-value for the interaction likelihood ratio test was *p* = *0.0*01). Meanwhile, it was not significant in the low magnesium intake group (*p* = 0.225). When vitamin D was transformed into a categorical variable, there was still an interaction between vitamin D and the incidence of diabetes both in participants with low magnesium intake and those with high magnesium intake (the *p*-value for the interaction likelihood ratio test was *p* < 0.001). In the high magnesium intake group, the weighted odds ratio for T2D of sufficient vitamin D group was 0.647 (95%CIs: 0.462–0.907, *p* = 0.015) compared with the reference group (vitamin D deficiency group). Meanwhile, the difference was not significant in the low magnesium intake group (OR = 0.877 95%CIs: 0.596–1.289, *p* = 0.507). The OR value, β value, and *P*-value of confounders in the weighted logistic regression model were shown in Supplementary Tables S4, S5.

We also performed a sensitivity analysis to robust our findings. After excluding the participants whose serum vitamin D value was >143.2 nmol/L, the results remained stable (Supplementary Table S6). As total vitamin D increased, the incidence of T2D in the high magnesium intake group was significantly reduced (OR = 0.878, 95%CIs: 0.878–0.981, *p* = 0.011), while the reduction was not significant in the low magnesium intake group (OR = 0.981, 95%CIs: 0.921–1.045, and *p* = 0.560). The interaction of magnesium intake on the association of vitamin D and T2D incidence was also significant (the *p*-value for the interaction likelihood ratio test was *p* < 0.001). When vitamin D was transformed into a categorical variable, the interaction still existed (the *p*-value for the interaction likelihood ratio test was *p* < 0.001). When participants whose serum vitamin D value was <9.2 nmol/L or >114.7 nmol/L were excluded from the analysis (Supplementary Table S7), as total vitamin D increased, a significant reduction in the incidence of T2D was observed in the high magnesium intake group (OR = 0.934, 95%CIs:0.877–0.995, and *p* = 0.04). However, the reduction was not significant in the low magnesium intake group (OR = 0.979, 95% CIs: 0.919–1.044, and *p* = 0.526). The *p*-value for the interaction likelihood ratio test was *p* = 0.002.

## Discussion

In this nationally representative sample of adults over 20 years of age, our findings indicated that among participants with high magnesium intake, serum vitamin D level was significantly higher in non-T2D participants compared with T2D participants. Serum vitamin D concentration was inversely associated with the risk of T2D in participants with high magnesium intake but not in the low magnesium intake group. Furthermore, an interactive effect between magnesium dietary intake and vitamin D on T2D was found, which indicated that vitamin D sufficiency and high magnesium intake are greater than the sum of the individual effects.

Studies have shown that vitamin D plays a role in T2D prevention by improving insulin secretion, insulin sensitivity, and suppressing systemic inflammation (19). Supportive of our results, in two prospective cohort studies (7, 20), the results indicated that a higher 25(OH)D concentration had a negative association with the risk of T2D. Moreover, a Mendelian randomization study of 96,423 white Danes suggested that participants with a reduction in plasma 25(OH)D concentration of 20 nmol/L also had an increased risk of T2D (21). Song et al. (22) conducted a meta-analysis which showed that a 38% decreased risk of developing T2D was observed in the highest tertile of 25OHD compared with the lowest tertile (RR 0.62, 95% CI = 0.54–0.7) (22).

However, a randomized, double-blind, placebo-controlled clinical trial in America did not find any effect of vitamin D supplements on the risk of T2D (23). Moreover, in the Nurses' Health Study conducted in America, the association between total vitamin D intake and T2D became non-significant after adjusting for dietary factors (8). One previous study indicated that the average magnesium intake in Americans was lower than RDA (13), which might explain the nonsignificant association between vitamin D and T2D risk. A cross-sectional study among the Thai population found an association between vitamin D and risk of T2D only in the urban elderly but not in rural residents or young urban residents (24).

Magnesium acts as a cofactor for more than 600 enzymes and an activator for an additional 200 (25). Magnesium deficiency may impair insulin secretion because of dysfunction of the ATP-sensitive K^+^ channels, which will disrupt the normal functioning of beta cells (26). Rosolova et al. (27) reported that subjects with a low concentration of plasma Mg^2+^ have a high concentration of fasting plasma insulin. Besides, magnesium is vital for the phosphorylation of insulin receptors and the activity of other signal kinases in the insulin signaling pathway (26). In addition, magnesium may directly affect glucose transporter protein activity 4 (GLUT4) and help regulate glucose translocation into the cell (28). Studies have found that consumption of magnesium-rich food may reduce T2D risk (29, 30), and an inverse association of magnesium intake with diabetes incidence was found in a long-term prospective study (31).

We believe our findings are biologically plausible. Vitamin D in circulation is biologically inactive. The activation of vitamin D occurs as follows: first, the circulating vitamin D is bound to the vitamin D-binding protein (VDBP) and transported to the liver where it gets hydroxylated to 25-hydroxyvitamin D [25(OH)D] by the enzyme 25-hydroxylase. Thereafter, [25(OH)D] is converted to 1,25-dihydroxyvitamin D [1,25(OH)2D], which is the biologically active form of vitamin D, by the enzyme 1α hydroxylase primarily in the kidney (32). It is noteworthy that magnesium is a crucial cofactor for the enzymes 25-hydroxylase and 1α hydroxylase, and the activity of VDBP is also a magnesium-dependent process (32). Therefore, high magnesium may activate 25(OH)D and 1,25(OH)2D synthesis and increase the transfer to the target tissue. Besides, as Swaminathan R reported, 1,25(OH)2D improves the intestinal absorption of magnesium (33).

On this basis, an NHANES study suggested that a high magnesium intake is significantly related to decreased risk of vitamin D insufficiency and deficiency (34). Besides, a randomized trial suggested that magnesium sufficiency may be necessary for optimizing vitamin D levels. The interaction between magnesium treatment and plasma vitamin D is also significant in this study (35). Wesselink (36) found an effect of the interaction between magnesium intake and vitamin D status on the risk of mortality in colorectal patients. They suggested that vitamin D sufficiency combined with an adequate magnesium intake was essential to reduce the risk of mortality in colorectal cancer patients (36).

There are some limitations to this study. First, as a cross-sectional observation study, the associations found in this study may not result in direct causality and could be confounded by some other unmeasured variables. However, several potential confounders including some dietary factors were adjusted in the logistic regression model. Second, although we used a large sample, the study population was limited to US residents. Therefore, due consideration of this aspect is necessary when extrapolating to other populations. Third, recall and self-reporting bias might occur, as dietary data were obtained from self-reported 24-h dietary recalls. If resampled in a different circle, the same participant may lead to an inaccurate result. However, the probability of such an eventuality is very small because NHANES examined about 5,000 people in 15 different counties across the country each year, and the U.S. population is about 327 million people. Besides, the participants were selected using a multistage, stratified probability design. Finally, compared with the excluded participants (Supplementary Table S8), the enrolled participants seemed to be younger. However, most baseline characteristics have no significant difference and most of the related variables have been included in the regression models. Nevertheless, given these limitations, well-designed multi-center-controlled trials are essential to verify our findings.

## Conclusions

In conclusion, the present study found that magnesium intake may affect the association between vitamin D and T2D. Although we had provided some clinical clues, further randomized controlled studies are required to provide more evidence.

## Data Availability Statement

The original contributions presented in the study are included in the article/Supplementary Material, further inquiries can be directed to the corresponding author/s.

## Ethics Statement

The survey protocol for the NHANES was approved by CDC's National Center for Health Statistics Institutional Research Ethics Review Board. All participants provided written informed consent, and the study was approved by the NCHS Research Ethics Review Board (https://wwwn.cdc.gov/nchs/nhanes/default.aspx). Human subjects were not involved in this study.

## Author Contributions

WH conducted data collection, analysis, and wrote the manuscript. XM modified the manuscript. HLia conducted data collection. HLi conducted data interpretation. JC drew the figure. LF made the table. QY and ZZ designed the study and reviewed the manuscript. All authors contributed to the article and approved the submitted version.

## Funding

This study was supported by New Technology and New Clinical Research and Application Fund Projects of the Second Affiliated Hospital of Guangzhou Medical University (2018-XJS-Y-02 and 2020-LCYJ-XJS-08).

## Conflict of Interest

The authors declare that the research was conducted in the absence of any commercial or financial relationships that could be construed as a potential conflict of interest.

## Publisher's Note

All claims expressed in this article are solely those of the authors and do not necessarily represent those of their affiliated organizations, or those of the publisher, the editors and the reviewers. Any product that may be evaluated in this article, or claim that may be made by its manufacturer, is not guaranteed or endorsed by the publisher.

## Abbreviations

T2D: type 2 diabetes
BMI: body mass index.
