# Supplementary material for: Dietary Magnesium Intake Affects the Association Between Serum Vitamin D and Type 2 Diabetes: A Cross-Sectional Study
Source: Front Nutr. 2021 Nov 25;8:763076. doi: 10.3389/fnut.2021.763076 (PMC8656460; doi:10.3389/fnut.2021.763076)
Supplement: Supplementary file 1 [file Data_Sheet_1.docx]

| TABLE S1 Interactive effect of vitamin D and dietary magnesium intake on T2D (Model 1) | | | | | | |
| --- | --- | --- | --- | --- | --- | --- |
| Variable | Low-magnesium intake | |  | High-magnesium intake | | *P* for interaction |
|  | (n=5,094) | |  | (n=5,155) | |  |
|  | OR(95%CI) | *P* value |  | OR(95%CI) | *P* value |  |
| Vitamin D (nmol/dL) | 0.964 (0.925-1.003) | 0.078 |  | 0.926 (0.883-0.971) | 0.002 | <0.001 |
| Deficient (<50nmol/L) | 1(reference) |  |  | 1(reference) |  | <0.001 |
| Suboptimal (50-75nmol/L) | 0.770 (0.597-0.993) | 0.048 |  | 0.668 (0.512-0.873) | 0.004 |  |
| Sufficient (>75nmol/L) | 0.820 (0.625-1.078) | 0.161 |  | 0.643 (0.470-0.880) | 0.008 |  |
| Trend test |  | 0.172 |  |  | 0.018 |  |
| No covariate was adjusted. | | | | | | |

| Table S2 Interactive effect of vitamin D and dietary magnesium intake on T2D (Model 2) | | | | | | |
| --- | --- | --- | --- | --- | --- | --- |
| Variable | Low-magnesium intake | |  | High-magnesium intake | | *P* for interaction |
|  | (n=5,094) | |  | (n=5,155) | |  |
|  | OR(95%CI) | *P* value |  | OR(95%CI) | *P* value |  |
| Vitamin D (nmol/dL) | 0.962 (0.917-1.010) | 0.123 |  | 0.918 (0.874-0.964) | 0.001 | <0.001 |
| Subgroups | |  |  |  |  | <0.001 |
| Deficient (<50nmol/L) | 1(reference) |  |  | 1(reference) |  |  |
| Suboptimal (50-75nmol/L) | 0.821 (0.585-1.151) | 0.257 |  | 0.663 (0.498-0.882) | 0.006 |  |
| Sufficient (>75nmol/L) | 0.847 (0.598-1.199) | 0.353 |  | 0.614 (0.433-0.869) | 0.008 |  |
| Trend test |  | 0.367 |  |  | 0.157 |  |
| Adjusted for age, gender, race/ethnicity. | | | | | | |
|  |  |  |  |  |  |  |
|  |  |  |  |  |  |  |
|  |  |  |  |  |  |  |

| TABLE S3 Interactive effect of vitamin D and dietary magnesium intake on T2D (Model 3) | | | | | | |
| --- | --- | --- | --- | --- | --- | --- |
| Variable | Low-magnesium intake | |  | High-magnesium intake | | *P* for interaction |
|  | (n=5,094) | |  | (n=5,155) | |  |
|  | OR(95%CI) | *P* value |  | OR(95%CI) | *P* value |  |
| Vitamin D (nmol/dL) | 0.967(0.919-1.019) | 0.213 |  | 0.926 (0.883-0.971) | 0.003 | 0.001 |
| Subgroups | | |  |  |  | <0.001 |
| Deficient (<50nmol/L) | 1(reference) |  |  | 1(reference) |  |  |
| Suboptimal (50-75nmol/L) | 0.842 (0.590-1.201) | 0.347 |  | 0.688 (0.521-0.908) | 0.011 |  |
| Sufficient (>75nmol/L) | 0.872 (0.597-1.274) | 0.483 |  | 0.653 (0.465-0.916) | 0.017 |  |
| Trend test |  | 0.495 |  |  | 0.031 |  |
| Adjusted for age, gender, race/ethnicity, BMI, education level, physical activity, smoking status, PIR, season of examination. | | | | | | |

| TABLE S4 OR value and 95% CIs of confounders in weighted logistic regression model in low magnesium intake group | | | |
| --- | --- | --- | --- |
| Variable | Low-magnesium intake | | |
|  | (n=5,094) | | |
|  | OR (95%CI) | β (95%CI) | *P* |
| Vitamin D (nmol/L) | 0.968 (0.919-1.020) | -0.033 (-0.084 ~ 0.020) | 0.225 |
| Age (years) | 1.065 (1.059-1.072) | 0.063 (0.057 ~ 0.070) | <0.001 |
| Gender (%) |  |  |  |
| Male | 1(reference) |  |  |
| Female | 0.699 (0.570-0.857) | -0.358 (-0.562 ~ -0.154) | 0.001 |
| Race/ethnicity (%) |  |  |  |
| Mexican American | 1(reference) |  |  |
| Other Hispanic | 0.969 (0.574-1.634) | -0.032 (-0.555 ~ 0.491) | 0.906 |
| Non-Hispanic white | 0.541 (0.385-0.760) | -0.614 (-0.955 ~ -0.274) | <0.001 |
| Non-Hispanic black | 0.913 (0.652-1.279) | -0.091 (-0.428 ~ 0.246) | 0.6 |
| Other races | 1.074 (0.655-1.762) | 0.071 (-0.423 ~ 0.566) | 0.779 |
| Obesity (%) |  |  |  |
| No | 1(reference) |  |  |
| Yes | 3.629 (2.887-4.563) | 1.289 (1.060 ~ 1.518) | <0.001 |
| Education level (%) |  |  |  |
| Did not graduate from high school | 1(reference) |  |  |
| Graduated from high school | 0.885 (0.642-1.221) | -0.157 (-0.443 ~ 0.200) | 0.463 |
| College education or above | 0.976 (0.748-1.275) | -0.024 (-0.290 ~ 0.243) | 0.861 |
| Physical activity (%) |  |  |  |
| Vigorous work activity | 1(reference) |  |  |
| Moderate work activity | 1.214 (0.808-1.823) | 0.194 (-0.213 ~ 0.600) | 0.356 |
| Walk or bicycle | 1.809 (1.170-2.798) | 0.593 (0.157 ~ 1.029) | 0.011 |
| Vigorous recreational activities | 1.620 (0.951-2.761) | 0.482 (-0.050 ~ 1.016) | 0.083 |
| Moderate recreational activities | 1.052 (0.757-1.462) | 0.051 (-0.278 ~ 0.380) | 0.764 |
| Smoking status (%) |  |  |  |
| Current smoker | 1(reference) |  |  |
| Former smoker | 1.286 (0.901-1.835) | 0.252 (-0.104 ~ 0.607) | 0.173 |
| Never smoker | 1.235 (0.929-1.641) | 0.211 (-0.074 ~ 0.495) | 0.154 |
| Season of examination (%) |  |  |  |
| Winter | 1(reference) |  |  |
| Summer | 1.017 (0.796-1.298) | 0.017 (-0.228 ~0.261) | 0.896 |
| PIR (%) | 0.907 (0.834-0.986) | -0.098 (-0.182 ~ -0.014) | 0.026 |
| Dietary calcium intake (mg) | 0.99991 (0.99953-1.00028) | -0.00009 (-0.00047 ~ 0.00028) | 0.633 |
| Adjusted for age, gender, race/ethnicity, obesity, education level, physical activity, smoking status, PIR, season of examination, dietary calcium intake. | | | |

| TABLE S5 OR value and 95% CIs of confounders in weighted logistic regression model in high magnesium intake group | | | |
| --- | --- | --- | --- |
| Variable | High-magnesium intake | | |
|  | (n=5,155) | | |
|  | OR (95%CI) | β (95%CI) | *P* |
| Vitamin D (nmol/L) | 0.925 (0.883-0.970) | -0.078 (-0.124 ~ 0.030) | 0.002 |
| Age (years) | 1.065 (1.056-1.075) | 0.063 (0.054 ~ 0.072) | <0.001 |
| Gender (%) |  |  |  |
| Male | 1(reference) |  |  |
| Female | 0.728 (0.574-0.923) | -0.318 (-0.555 ~ -0.080) | 0.012 |
| Race/ethnicity (%) |  |  |  |
| Mexican American | 1(reference) |  |  |
| Other Hispanic | 1.127 (0.726-1.751) | 0.120 (-0.320 ~ 0.560) | 0.597 |
| Non-Hispanic white | 0.711 (0.508-0.994) | -0.341 (-0.677 ~ -0.006) | 0.052 |
| Non-Hispanic black | 0.985 (0.685-1.417) | -0.015 (-0.378 ~ 0.349) | 0.936 |
| Other races | 1.854 (1.122-3.061) | 0.617 (0.115 ~ 1.119) | 0.02 |
| Obesity (%) |  |  |  |
| No | 1(reference) |  |  |
| Yes | 3.695 (2.871-4.754) | 1.307 (1.055 ~ 1.559) | <0.001 |
| Education level (%) |  |  |  |
| Did not graduate from high school | 1(reference) |  |  |
| Graduated from high school | 0.737 (0.513-1.059) | -0.305 (-0.667 ~ 0.057) | 0.106 |
| College education or above | 0.546 (0.383-0.779) | -0.605 (-0.960 ~ -0.250) | 0.002 |
| Physical activity (%) |  |  |  |
| Vigorous work activity | 1(reference) |  |  |
| Moderate work activity | 1.372 (0.925-2.036) | 0.316 (-0.078 ~ 0.711) | 0.123 |
| Walk or bicycle | 1.625 (1.074-2.459) | 0.486 (0.071 ~ 0.900) | 0.026 |
| Vigorous recreational activities | 1.718 (0.894-3.305) | 0.541 (-0.112 ~ 1.198) | 0.112 |
| Moderate recreational activities | 1.282 (0.880-1.867) | 0.248 (-0.128 ~ 0.624) | 0.203 |
| Smoking status (%) |  |  |  |
| Current smoker | 1(reference) |  |  |
| Former smoker | 1.067 (0.702-1.621) | 0.065 (0.354 ~ 0.483) | 0.764 |
| Never smoker | 1.041 (0.771-1.406) | 0.040 (-0.260 ~ 0.341) | 0.796 |
| Season of examination (%) |  |  |  |
| Winter | 1(reference) |  |  |
| Summer | 0.950 (0.725-1.243) | -0.051 (-0.322 ~ 0.218) | 0.708 |
| PIR (%) | 0.941 (0.876-1.010) | -0.061 (-0.132 ~ 0.001) | 0.1 |
| Dietary calcium intake (mg) | 1.00007 (0.99983-1.00030) | 0.00007 (-0.00017 ~ 0.00030) | 0.582 |
| Adjusted for age, gender, race/ethnicity, obesity, education level, physical activity, smoking status, PIR, season of examination, dietary calcium intake. | | | |

| TABLE S6 Interactive effect of vitamin D and dietary magnesium intake on T2D (Participants whose serum vitamin D value greater than 143.2 nmol/L were excluded) | | | | | | |
| --- | --- | --- | --- | --- | --- | --- |
| Variable | Low-magnesium intake | |  | High-magnesium intake | | *P* for interaction |
|  | (n=5,034) | |  | (n=5,098) | |  |
|  | OR(95%CI) | *P* value |  | OR(95%CI) | *P* value |  |
| Vitamin D (nmol/dL) | 0.981 (0.921-1.045) | 0.560 |  | 0.928 (0.878-0.981) | 0.011 | <0.001 |
| Subgroups | | |  |  |  | <0.001 |
| Deficient (<50nmol/L) | 1(reference) |  |  | 1(reference) |  |  |
| Suboptimal (50-75nmol/L) | 0.858 (0.604-1.219) | 0.396 |  | 0.686 (0.523-0.901) | 0.010 |  |
| Sufficient (>75nmol/L) | 0.913 (0.619-1.345) | 0.647 |  | 0.665 (0.472-0.937) | 0.024 |  |
| Trend test |  | 0.655 |  |  | 0.043 |  |
| Adjusted for age, gender, race/ethnicity, BMI, education level, physical activity, smoking status, PIR, season of examination and dietary calcium intake. | | | | | | |

| TABLE S7 Interactive effect of vitamin D and dietary magnesium intake on T2D (Participants whose serum vitamin D level greater than 118.7 nmol/L or less than 9.2 nmol/L were excluded) | | | | | | |
| --- | --- | --- | --- | --- | --- | --- |
| Variable | Low-magnesium intake | |  | High-magnesium intake | | *P* for interaction |
|  | (n=4,905) | |  | (n=4,951) | |  |
|  | OR(95%CI) | *P* value |  | OR(95%CI) | *P* value |  |
| Vitamin D (nmol/dL) | 0.979 (0.919-1.044) | 0.526 |  | 0.934 (0.877-0.995) | 0.040 | 0.002 |
| Subgroups | | |  |  |  | 0.002 |
| Deficient (<50nmol/L) | 1(reference) |  |  | 1(reference) |  |  |
| Suboptimal (50-75nmol/L) | 0.828 (0.579-1.185) | 0.308 |  | 0.672 (0.511-0.884) | 0.007 |  |
| Sufficient (>75nmol/L) | 0.903 (0.622-1.311) | 0.595 |  | 0.677 (0.477-0.961) | 0.034 |  |
| Trend test |  | 0.596 |  |  | 0.060 |  |
| \| TABLE S8 Baseline characteristics of excluded participants and enrolled participants \| \| \| \| \| \| --- \| --- \| --- \| --- \| --- \| \| Covariates \| Total (n = 20,655) \| excluded participants (n = 10,406) \| enrolled participants (n = 10,249) \| *p*-value \| \| Age (years) \| 49.5 ± 17.7 \| 50.0 ± 17.8 \| 49.0 ± 17.7 \| < 0.001 \| \| Gender, n (%) \|  \|  \|  \| 0.848 \| \| Male \| 10027 (48.5) \| 5059 (48.6) \| 4968 (48.5) \|  \| \| Female \| 10628 (51.5) \| 5347 (51.4) \| 5281 (51.5) \|  \| \| Race/Ethnicity, n (%) \|  \|  \|  \| < 0.001 \| \| Mexican America \| 3074 (14.9) \| 1604 (15.4) \| 1470 (14.3) \|  \| \| Other Hispanic \| 2092 (10.1) \| 1087 (10.4) \| 1005 (9.8) \|  \| \| Non-Hispanic white \| 9226 (44.7) \| 4488 (43.1) \| 4738 (46.2) \|  \| \| Non-Hispanic black \| 4168 (20.2) \| 2103 (20.2) \| 2065 (20.1) \|  \| \| Other races \| 2095 (10.1) \| 1124 (10.8) \| 971 (9.5) \|  \| \| BMI(kg/m2), Mean ± SD \| 29.0 ± 6.9 \| 29.0 ± 6.9 \| 29.1 ± 6.9 \| 0.712 \| \| Obesity, n (%) \|  \|  \|  \| 0.629 \| \| No \| 12802 (62.7) \| 6389 (62.9) \| 6413 (62.6) \|  \| \| Yes \| 7603 (37.3) \| 3767 (37.1) \| 3836 (37.4) \|  \| \| Season of examination, n (%) \|  \|  \|  \| 0.401 \| \| Winter \| 9636 (46.7) \| 4824 (46.4) \| 4812 (47) \|  \| \| Summer \| 11019 (53.3) \| 5582 (53.6) \| 5437 (53) \|  \| \| Two Hour Glucose (OGTT) (mmol/L) \| 6.7 ± 2.9 \| 6.8 ± 3.0 \| 6.7 ± 2.9 \| 0.049 \| \| Fasting Glucose (mmol/L) \| 6.0 ± 2.0 \| 6.1 ± 2.0 \| 6.0 ± 1.9 \| 0.002 \| \| Glycohemoglobin (%) \| 5.8 ± 1.1 \| 5.8 ± 1.1 \| 5.7 ± 1.1 \| 0.002 \| \| Smoking status, n (%) \|  \|  \|  \| 0.254 \| \| current smoker \| 2572 (21.4) \| 370 (21) \| 2202 (21.5) \|  \| \| former smoker \| 2883 (24.0) \| 400 (22.7) \| 2483 (24.2) \|  \| \| never smoker \| 6556 (54.6) \| 992 (56.3) \| 5564 (54.3) \|  \| \| Physical activity, n (%) \|  \|  \|  \| < 0.001 \| \| Vigorous work activity \| 2391 (16.5) \| 555 (13.1) \| 1836 (17.9) \|  \| \| Moderate work activity \| 3212 (22.2) \| 1055 (24.9) \| 2157 (21) \|  \| \| Walk or bicycle \| 2439 (16.8) \| 998 (23.6) \| 1441 (14.1) \|  \| \| Vigorous recreational activities \| 1246 (8.6) \| 564 (13.3) \| 682 (6.7) \|  \| \| Moderate recreational activities \| 5194 (35.9) \| 1061 (25.1) \| 4133 (40.3) \|  \| \| Education level, n (%) \|  \|  \|  \| < 0.001 \| \| Did not graduate from high school \| 5360 (26.0) \| 2797 (26.9) \| 2563 (25) \|  \| \| Graduated from high school \| 4683 (22.7) \| 2399 (23.1) \| 2284 (22.3) \|  \| \| College education or above \| 10588 (51.3) \| 5195 (50) \| 5393 (52.7) \|  \| \| PIR, n (%) \|  \|  \|  \| 0.276 \| \| <1 \| 4246 (22.4) \| 1995 (22.9) \| 2251 (22) \|  \| \| 1-3 \| 7830 (41.3) \| 3563 (40.9) \| 4267 (41.6) \|  \| \| >3 \| 6876 (36.3) \| 3145 (36.1) \| 3731 (36.4) \|  \| \| Dietary factors \|  \|  \|  \|  \| \| Energy (kcal) \| 2098.6 ± 1001.2 \| 2071.1 ± 983.7 \| 2123.0 ± 1015.9 \| < 0.001 \| \| Protein(gm) \| 73.4 (52.0, 100.1) \| 72.8 (51.4, 99.0) \| 73.9 (52.6, 101.1) \| 0.008 \| \| Fiber(gm) \| 14.5 (9.5, 21.3) \| 14.5 (9.4, 21.3) \| 14.5 (9.6, 21.3) \| 0.446 \| \| Magnesium(mg) \| 267.0 (194.0, 363.0) \| 267.0 (193.0, 361.0) \| 267.0 (194.0, 364.0) \| 0.094 \| \| Calcium(mg) \| 807.0 (523.0, 1178.0) \| 803.0 (519.0, 1171.0) \| 809.0 (528.0, 1182.0) \| 0.221 \| \| Vitamin D(mg) \| 3.2 (1.3, 6.0) \| 3.2 (1.3, 6.1) \| 3.1 (1.2, 6.0) \| 0.643 \| \| Risk for diabetes \|  \|  \|  \| 0.547 \| \| Yes \| 2313 (12.8) \| 1142 (12.6) \| 1171 (13) \|  \| \| No \| 15723 (86.9) \| 7884 (87.1) \| 7839 (86.7) \|  \| \| Unknown \| 50 (0.3) \| 22 (0.2) \| 28 (0.3) \|  \| \| T2D, n (%) \|  \|  \|  \| < 0.001 \| \| No \| 16827 (81.5) \| 8380 (80.5) \| 8447 (82.4) \|  \| \| Yes \| 3828 (18.5) \| 2026 (19.5) \| 1802 (17.6) \|  \| \| PIR, Ratio of family income to poverty; BMI, Body Mass Index; T2D, type 2 diabetes. \| \| \| \| \|   Adjusted for age, gender, race/ethnicity, BMI, education level, physical activity, smoking status, PIR, season of examination and dietary calcium intake. | | | | | | |


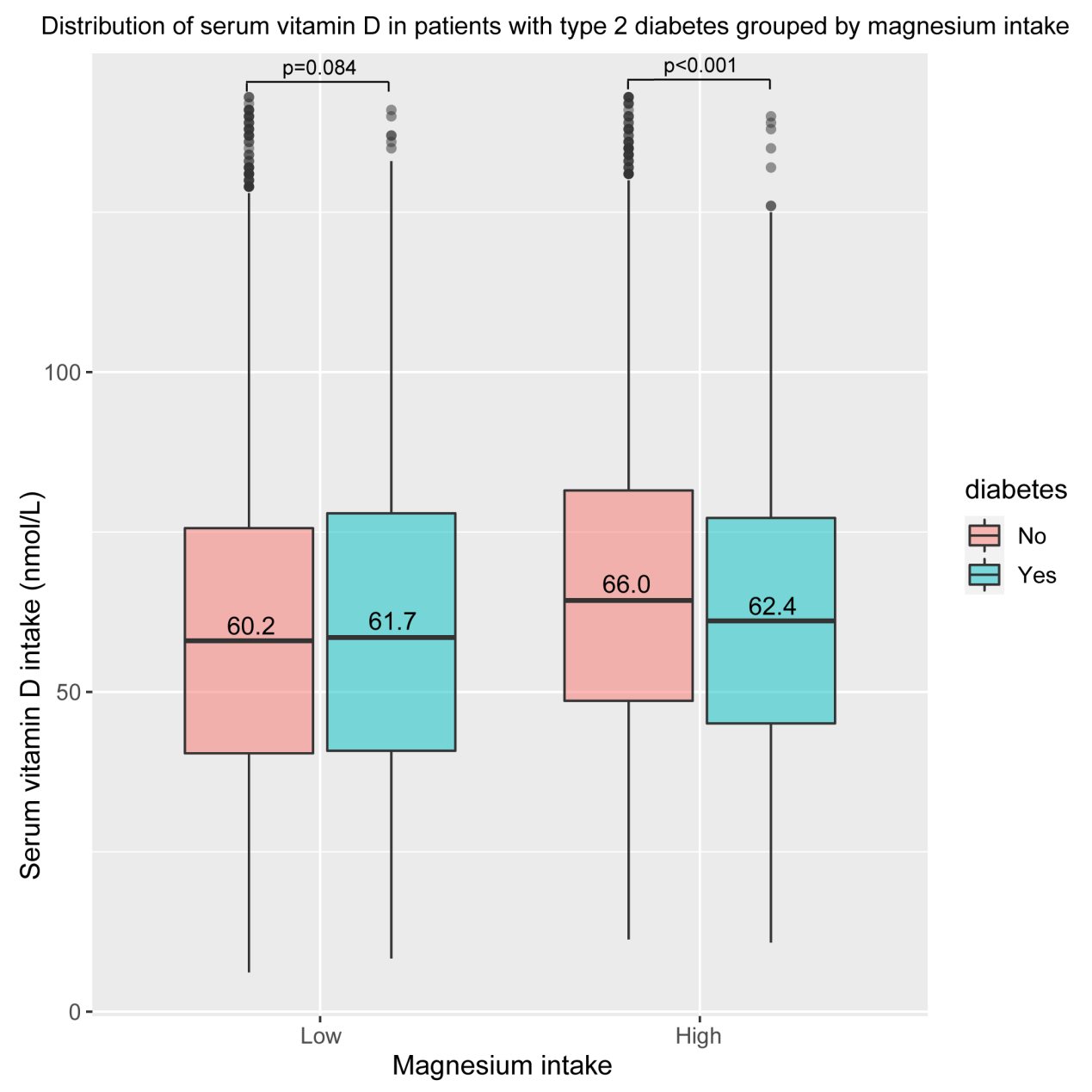


Figure S1. Distribution of serum vitamin D in patients with type 2 diabetes grouped by magnesium intake (participants whose serum vitamin D value greater than 143.2 nmol/L were excluded).


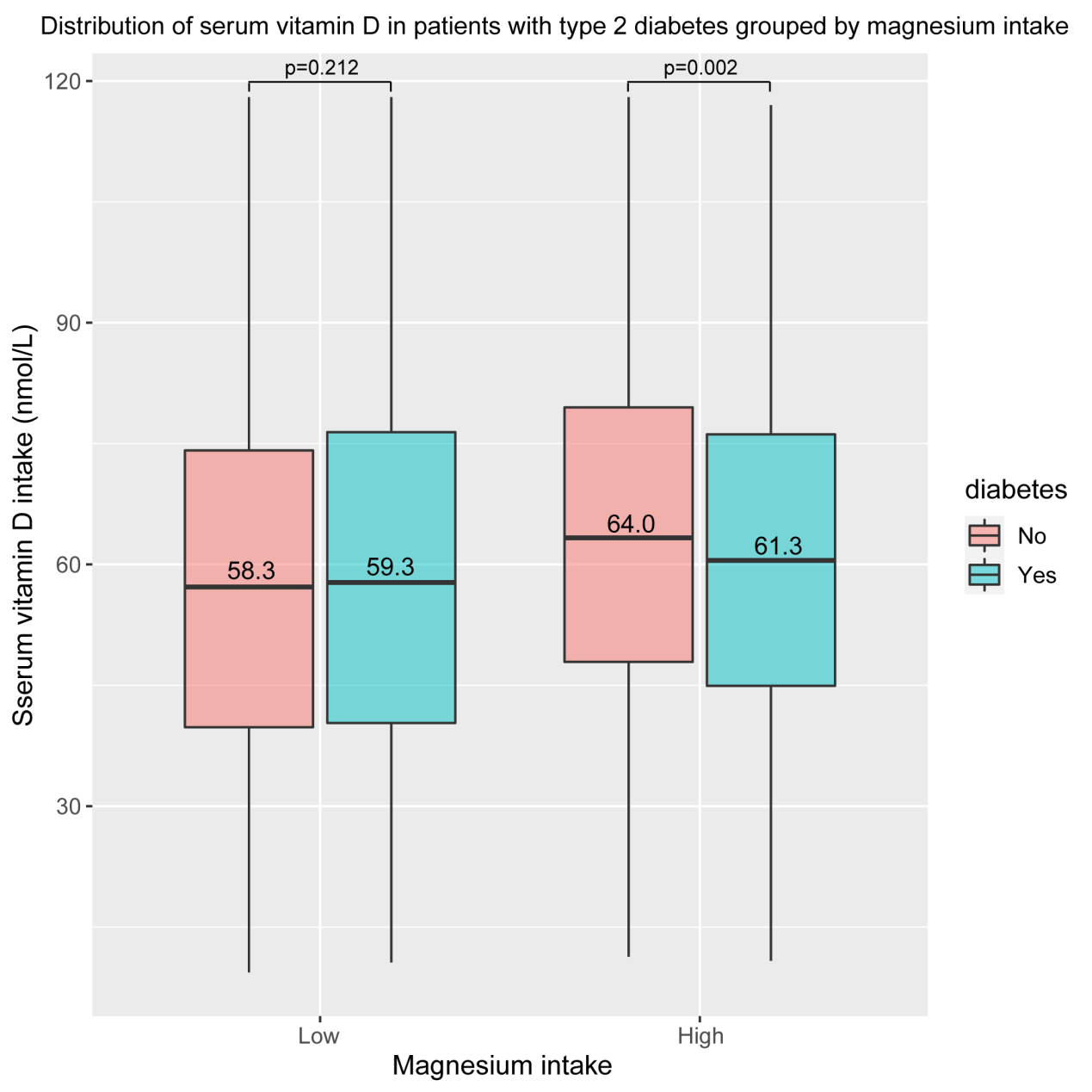


Figure S2: Distribution of serum vitamin D in patients with type 2 diabetes grouped by

magnesium intake (Participants whose serum vitamin D value less than 9.2 or greater than 118.7

were excluded).
